# Improving medication management for patients with multimorbidity in primary care: a qualitative feasibility study of the MY COMRADE implementation intervention

**DOI:** 10.1186/s40814-017-0129-8

**Published:** 2017-03-20

**Authors:** Carol Sinnott, Molly Byrne, Colin P. Bradley

**Affiliations:** 1 Department of General Practice, Western Gateway Building, University College Cork, Cork, Ireland; 2 Health Behaviour Change Research Group, School of Psychology, National University of Ireland, Galway, Ireland

## Abstract

**Background:**

For the majority of patients with multimorbidity, the prescription of multiple long-term medications (polypharmacy) is indicated. However, polypharmacy poses a risk of adverse drug events, drug interactions and excessive treatment burdens. To help general practitioners (GPs) conduct more comprehensive medication reviews for patients with multimorbidity, we developed the theoretically-informed MultimorbiditY COllaborative Medication Review And DEcision Making (MY COMRADE) implementation intervention. In this study, we assessed the feasibility and acceptability of MY COMRADE by GPs.

**Methods:**

A non-randomised feasibility study using a qualitative framework approach was conducted. General practices were recruited by purposively sampling from interested GPs attending continuing professional development meetings (CPD) in southwest Ireland. Participating practices were instructed on the MY COMRADE implementation intervention which has five components: (i) action planning; (ii) allocation of protected time; (iii) peer-supported medication review; (iv) use of a prescribing checklist and (v) self-incentives (allocation of CPD points). GPs in participating practices agreed to conduct medication reviews on multimorbid patients from their own caseload using the MY COMRADE approach. After completing these reviews, qualitative interviews were conducted to evaluate GPs’ experiences of the intervention and were analysed using the framework method.

**Results:**

GPs from ten practices participated in the study. The GPs reported that MY COMRADE was an acceptable approach to implementing medication review in general practice, especially for complex patients with multimorbidity. Action plans for the medication reviews varied between practices, but all reviews led to recommendations for optimising medications and patient safety. Many GPs felt that using the MY COMRADE approach would ultimately lead to more efficient use of their time, but a minority felt that the time and cost implications of using two GPs to review medications would not be sustainable unless greater incentives were used.

**Conclusions:**

This study demonstrates that MY COMRADE is an acceptable and feasible approach to supporting comprehensive medication reviews for patients with multimorbidity. These findings indicate that a large scale trial of the effectiveness of MY COMRADE is now required to fully evaluate its potential to change prescribing behaviour and improve downstream outcomes such as prescribing appropriateness and treatment burden.

**Trial registration:**

ISRCTN registry: ISRCTN34837446.

**Electronic supplementary material:**

The online version of this article (doi:10.1186/s40814-017-0129-8) contains supplementary material, which is available to authorized users.

## Background

Internationally, healthcare policy makers strive to deliver generalist management of chronic disease in a primary care setting [1]. Over 50% of patients with chronic disease have multimorbidity (multiple chronic diseases) [2], which can lead to challenges in the provision of clinical care foremost of which is the management of multiple medications [3]. Multimorbidity is associated with higher rates of potentially inappropriate prescribing and adverse drug effects [4]; therefore, it is recommended that patients with multimorbidity have their medications reviewed periodically [5]. However, uncertainty about how to balance guideline adherence and minimising the negative effects of polypharmacy can deter primary care physicians or general practitioners (GPs) from actively reviewing medications for their multimorbid patients [6, 7]. As the prevalence of multimorbidity continues to rise, interventions to support structured medication review for patients with multimorbidity are a priority [8].

Existing approaches to enhancing medication review in general practice incorporate pharmacists [9], geriatricians [10] or clinical decision support systems [11]. Systematic reviews of the effects of these interventions have shown inconsistent results with only limited evidence to show that they reduce medication-related problems or lead to meaningful clinical improvements [9–11].

In response to these limitations, we developed a novel implementation intervention to support medication review by GPs for patients with multimorbidity. Implementation interventions are complex interventions that aim to align clinical behaviour with evidence-based practice [12]. The Medical Research Council UK (MRC) states that if such interventions are informed by empirical data and theory, they are easier to evaluate, more likely to be implemented and more likely to be worth implementing [13].

**Fig. 1:**
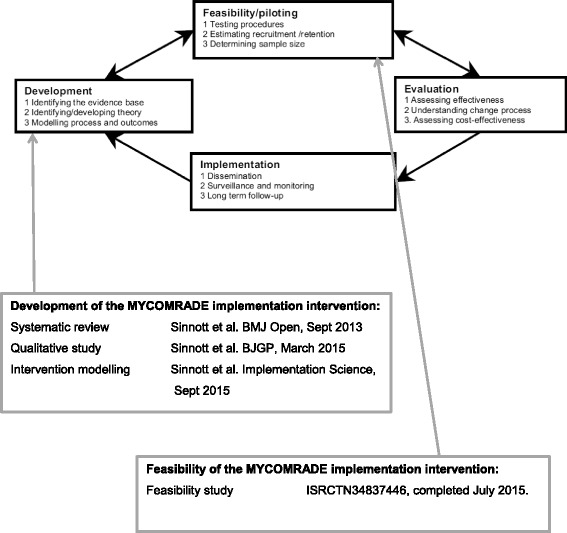
Key steps in the development and feasibility testing of the MY COMRADE intervention, following stages of the UK Medical Research Council guidance on the development and evaluation of complex interventions in healthcare [30]

We followed the guidance of the MRC by first conducting a synthesis of the existing evidence about GPs’ perceptions of managing multimorbidity (see Fig. 1) [14]. We added to this by conducting a qualitative interview study with GPs on medication management in multimorbidity [15]. We found that when the management of patients with multimorbidity gets complicated, GPs often seek advice from each other [15]. These discussions between GPs take place on an informal basis and are rarely documented in the medical notes, yet they represent an important source of peer support for decision-making in primary care. We developed an implementation intervention that takes advantage of these discussions by formalising them as structured medication reviews for patients with multimorbidity. We applied theories of behaviour (the COM-B), models of intervention design (the Behaviour Change Wheel) and taxonomies of behaviour change techniques to our empirical data to develop the Multimorbidity COllaborative Medication Review And DEcision Making (MY COMRADE) implementation intervention [16]. In the MY COMRADE implementation intervention, two GPs use protected time to conduct a structured medication review and generate an optimised medication management plan for a complex multimorbid patient together.

Although using empirical evidence and theoretical methods in the development of MY COMRADE increases its likelihood of success, the MRC guidance also stresses the importance of conducting feasibility and pilot studies of new interventions in real-world conditions prior to conducting larger and more costly evaluations of effectiveness. Feasibility and pilot studies can address key uncertainties, inform refinements and identify problems that might occur in an ensuing definitive trial of a complex intervention or can determine whether a full-scale study of an intervention is warranted [17]. In a framework of pilot and feasibility studies proposed by Eldridge et al. [17], a feasibility study for a future definitive randomised controlled trial asks whether the future trial can be done, should be done and, if so, how (see top box in Fig. 1). Pilot studies are a subset of feasibility studies: they ask the same questions about feasibility but have a particular design feature: in a pilot study (that might or might not be randomised), the future definitive trial, or part of it, is conducted on a smaller scale.

Therefore, prior to conducting a larger trial of MY COMRADE, we conducted this study to examine the feasibility of the MY COMRADE approach to implementing structured medication review for patients with multimorbidity. We assessed feasibility by examining three key uncertainties: is MY COMRADE acceptable to GPs; is it adaptable by GPs working in different contexts and do recommendations for medication optimisation arise from the medication reviews.

## Methods

### Study design

A non-randomised feasibility study of the MY COMRADE implementation intervention was conducted, using a qualitative framework approach.

### Setting and participants

The study was conducted between December 2014 and July 2015. General practices were recruited by purposively sampling from interested GPs attending continuing professional development meetings (CPD) in southwest Ireland. A brief description of the study was provided by one researcher (CS), and GPs who were interested signed a clipboard. From this list, practices were purposively sampled by location (urban/rural), practice size (≤3 GPs/>3 GPs) and GP training practice (yes/no). The only inclusion criterion was that practices have at least two qualified GPs. It was agreed a priori that ten practices manifesting this range of criteria would be sufficient to demonstrate feasibility [16]. The researcher (CS) visited each practice to describe the implementation intervention to GPs and to advise them on how they should proceed with using it. Written information and instruction leaflets were also provided (Additional files 1 and 2).

### The intervention

The development and specification of the implementation intervention are described in detail elsewhere [16]. In summary, MY COMRADE includes five behaviour change techniques (labelled below in italics). The principal behaviour change technique is peer support: two GPs conduct a structured medication review together to generate an optimised medication management plan for a complex multimorbid patient. The medication review is guided by a prescribing checklist (*prompts and cues*), which is provided to GPs in paper form and was adapted from the published “NO TEARS” medication review tool (Additional file 3) [18]. The other three behaviour change techniques are: (i) developing a practice action plan for conducting the reviews (*action planning*); (ii) allocating protected time within the GPs’ schedule to conduct the reviews (*restructuring of the social environment*) and (iii) recording the activity for the purposes of CPD points for GPs (*self-incentives*).

### Procedure

In each practice, GPs were asked to select complex multimorbid patients who were either prescribed ten or more medications or five or more medications with another complicating factor (i.e. impaired cognition, psychosocial complexity, poor life expectancy, etc.) from their caseload. They agreed to conduct medication reviews for at least six of these patients using the MY COMRADE approach. They were advised that each medication review would take approximately 10–15 min.

### Evaluation and outcomes

We evaluated GPs’ experiences of the intervention in semi-structured interviews after they had completed their medication reviews. The topic guide for the interviews was informed by the implementation outcome framework [19] [20] (topic guide provided in Additional file 4). This framework includes eight implementation outcomes (acceptability, adoption, appropriateness, feasibility, fidelity, implementation cost, coverage and sustainability) which we aligned with our research objectives. We also asked GPs to describe how they conducted their collaborative reviews and any recommendations on medications that arose in each review. We encouraged GPs to refer to notes they had made during the medication reviews to help them recount the details of each case.

### Analysis

All evaluation interviews were audio-recorded, transcribed verbatim and entered into NVivo software to facilitate data coding. Field notes and memos were also analysed. We used the framework approach for data analysis [21]. After familiarisation and immersion in the transcripts, sections of data were indexed into the eight headings of the implementation outcome framework, which were in turn mapped to the three specific research objectives. We mapped data on acceptability and adoption to the first research objective (i.e. if MY COMRADE was acceptable to GPs) and used data on the implementation cost and sustainability to develop a subtheme on longer term acceptability. We mapped feasibility and fidelity to the second research objective (i.e. adaptability by GPs working in different contexts) and mapped appropriateness and coverage to the third research objective (i.e. if recommendations for medication optimisation arose from the medication reviews). Inductive themes that did not fit with the a priori framework were added to the matrix. Themes and subthemes were compared across practices to map the range of experiences, provide explanations and find associations. All interviews were indexed, charted and analysed by CS, and three interviews were separately indexed and analysed by CB and MB. In a consensus meeting, all three researchers presented their analysis, discussed divergent accounts and refined emerging themes. The study was approved by the Clinical Research Ethics Committee, University College Cork (ECM3(vvvvv)19/11/14). The Template for Intervention Description and Replication (TIDIER) checklist was used to guide the study report.

## Results

From the GPs who expressed an interest in the study, 15 were contacted before ten practices agreed to participate (10/15, 66%). Practice characteristics are shown in Table 1.

**Table 1:** Characteristics of the practices participating in the feasibility study

	% of practices (*n*)
Practice location	
Rural	20 (2)
Urban	50 (5)
Mixed	30 (3)
Size of practice	
≤3 GPs	40 (4)
>3 GPs	60 (6)
GP training practice	
Yes	50 (5)
No	50 (5)

### Is MY COMRADE acceptable to GPs?

In all interviews, GPs reported positive experiences of the intervention. Many said it sounded acceptable at first hearing:

I felt I had something to gain and my patients had something to gain as well. I emailed you (back) very quickly because I was positive about the whole thing. gp4

Other GPs felt it would help them with patients they were worried about:

the minute we heard about it, patients pop up in your head-you know these ones who are on like 15 tablets and they are really complicated. gp11

The focus of the intervention on prescribing was a key factor:

The whole prescribing issue is a potential mine field… anything that concentrates my brain or helps me be a little bit more circumspect on what we are prescribing is a good thing. gp2

The use of peer support was viewed as acceptable because it was compatible with the GPs’ usual behaviour in practice:

There’s hardly a day goes by where I don’t say can I talk to you about this or she says can I talk to you about that gp9

#### Cost and sustainability

For GPs, the biggest perceived cost of MY COMRADE was time. The duration of the reviews (5 to 30 min) varied with the GPs’ knowledge of the patient, the number of medications prescribed and the number of problems exposed. Additional work was often generated by the reviews such as referral to specialists; contacting local pharmacists and multiple consultations with patients to discuss potential changes. The majority of GPs did not feel negatively about this work, seeing it as part of their job and potentially time saving in the end:

I wouldn’t really call it additional workload because if it’s in the patient’s interest its part of my work. gp4

Regarding sustainability, many GPs said they intended to continue using the intervention as it was *practical, useful and relevant* and had potential benefits for patient care. Others felt that external factors were needed to ensure it was sustained, such as financial remuneration or punitive measures (i.e. external audit of medications).

### How was the implementation intervention adapted by GPs?

We determined how GPs implemented and adapted the five behaviour change techniques incorporated into MY COMRADE, by asking about the feasibility of and fidelity to the intervention. The results are shown in Table 2. All participating practices implemented the intervention but as implementation took longer in some practices than others, we began to set specific dates for follow-up interviews. The imposed deadline may have led to fewer collaborative reviews in some practices, but it revealed the competing demands on GPs’ time as they tried to fit the intervention into existing practice. The median number of MY COMRADE reviews per GP pair was 4 (interquartile range 3–5.75).

**Table 2:** Implementation of the five behavioural change techniques in the MY COMRADE intervention by participating practices

	Practice number									
	1	2	3	4	5	6	7	8	9	10
Action planning	+	+	−	+	+	+	−	+	−	−
Restructuring social environment	+	+	+	+	+	+	+	+	−	+
Social support	+	+	+	+	+	+	+	+	+	+
Prompts and cues (checklist)	+	+	+	+	+	+	+	+	−	+
Self-incentives (CPD points)	−	+	+	+	−	+	−	+	−	−
Number of medication reviews completed	4	4	3	6	6	3	8	3	1	5

1. Action planning
  Action plans varied from agreeing to conduct the reviews before or after consultation sessions, to using time already allocated to non-consultation activities (i.e. practice meetings) for reviews. Three practices planned to use gaps in their schedules to conduct reviews opportunistically—this approach only worked if one of the GPs was championing the intervention (Practices 3, 7, 10).
2. Restructuring of the social environment
  GPs reported two benefits to conducting medication reviews outside of consultations. First, they could focus on the medications without being distracted by the patients’ presenting *crisis or catastrophe or issue with the hospital or something* gp14. Second, GPs liked going into the consultation already prepared for making suggestions, reporting that it was *easier to discuss it with someone else first* gp11.
3. Social support
  Articulating and justifying patients’ medications to another GP appeared to be the most important component of the implementation intervention. GPs who experimented with conducting reviews on their own (using only the checklist) reported that the collaborative approach was better as it revealed their prescribing “blind spots” and was often quicker than doing it alone. GPs adapted the intervention by conducting reviews with a pharmacist (Practice 1) or a specialist (Practice 9): this approach also led to recommendations for medication change and provided reassurance to GPs. Two GPs conducted reviews with patients: they reported these reviews took longer, were more confusing for the GP and did not generate the same clear actionable recommendations (Practice 5 and 7).
4. Prompts and cues
  All but one practice used the prescribing checklist in reviews. GPs reported that the checklist was necessary for giving early reviews as structure, but they referred to it less frequently as time went on. The checklist continued to be useful in cases where the GP had no pre-existing concerns about the medications, by directing and prompting review of the entire prescription.
5. Self-incentives
  Although gaining CPD points was not a primary motivating factor for participating GPs, they all reported that they would record the reviews for CPD purposes.

### What recommendations for medication optimisation arose from the reviews?

Every review led to recommendations for optimisation of medications. In many cases, GPs were shocked at the number of recommendations that arose:

It was amazing, took us right out of our comfort zone. I thought at the worst we would find one or two things that we might change … But in each of the cases, we were able to question about 50% of their actual meds gp9

The most common recommendation involved de-prescribing medications for which there was no clear indication (Practices 1, 3–7, 10), new evidence for use (Practices 4, 7, 8, 10) or a duplicate (Practices 1, 3, 4, 6–8). Recommendations for de-prescription most commonly involved bisphosphonates, high-dose proton pump inhibitors, statins and aspirin for primary prevention, long-term analgesics and benzodiazepines.

In some reviews, the recommendations included updating tests and vaccinations (Practices 1, 2, 4, 5, 7, 8), and ensuring patients were getting adequate follow-up from specialist teams (Practice 2, 4, 7, 8). The reviews led to updating patients’ records (Practices 1, 4–8, 10) and highlighting patient specific risks (i.e. allergies) (Practices 1, 4, 7). Indicated preventative medications were added or adjusted in Practices 1, 2, 4, 7, 9.

#### Discussing recommendations with patients

This was an inductive subtheme within the theme of recommendations arising from the reviews. GPs varied in their approach to discussing the recommendations with patients: some waited until the patient’s next consultation while others called patients in to discuss their recommendations. GPs said that patients reacted favourably to hearing their case had been discussed at a practice level. Most patients agreed to making the recommended changes but after discussion with the GP, a minority (*n* = 2) opted not to.

## Discussion

Prior to conducting a larger trial of MY COMRADE, we needed to demonstrate the feasibility of the intervention by addressing key uncertainties. Our key uncertainties relate to our three research objectives (is MY COMRADE acceptable to GPs; is it adaptable by GPs working in different contexts and do recommendations for medication optimisation arise from the medication reviews). By addressing these questions using a systematic approach and empirical evidence, the feasibility of the intervention has been confirmed.

Our findings show that an implementation intervention using protected time and peer support supported the conduct of comprehensive structured medication reviews. The intervention was acceptable to GPs; was readily adaptable by GPs working in different contexts and led to optimised medication management plans for all of the complex multimorbid patients that were reviewed. These findings are encouraging and indicate that proceeding to a larger scale trial evaluation of MY COMRADE is worthwhile. The study has also provided important information in preparation for the future trial. For example, we were interested in how GPs adapted the five behaviour change techniques in MY COMRADE to the context of their own practices [22]. Peer support appeared to be the key technique in generating recommendations for medication optimisation, but GPs innovated in where they found this support. While other professional sources (i.e. pharmacist, specialist) were reported to be useful, conducting the review with patients only was not: therefore, professional social support will be a compulsory component of any future iterations of the intervention. Insufficient time appeared to be the only reason participants did not do all six reviews, and many GPs felt that incentives are needed to support and sustain allocation of their time to this activity. This may not be intervention specific: when a system is over constrained with competing demands, as is the case in Irish general practice, the resources needed to make any new intervention succeed may be unavailable [23]. However, more substantial incentives may lever behaviour change and warrant consideration prior to embarking on a large scale evaluation of MY COMRADE.

The aim of MY COMRADE is to implement an evidence-based practice: medication review for patients with multimorbidity. Ultimately, we would like to show that MY COMRADE positively impacts on outcomes such as prescribing appropriateness and cost savings due to reductions in unnecessary medications. However, these outcomes are further down the causal pathway and are influenced by a host of other factors. We have not focused on outcomes down the causal pathway in this study to avoid underestimating the value of the implementation intervention to positively change behaviour in line with best practice [24].

### Comparison with other work

Many interventions to support medication review in primary care have used pharmacists (10–12). These interventions have shown inconsistent results and evidence of their impact on clinical outcomes is lacking. Furthermore, such approaches are not a pragmatic option in Irish healthcare where few publically funded community pharmacists exist [25]. Our study showed that the MY COMRADE implementation intervention enabled GPs to identify and rectify suboptimal prescribing without requiring input from external personnel (i.e. a pharmacist) other than robust, reliable and credible internet sources of prescribing information (i.e. the British National Formulary).

Alternative approaches to medication review utilise computer decision support or integration of multiple guidelines. These approaches do not incorporate professional judgement or provide the peer support that difficult clinical decisions often require in patients with multimorbidity [26]. In contrast, peer-supported reviews permit professional judgement, individualised care and can help maintain professional standards [27].

In MY COMRADE, two GPs undertake a structured medication review, and together, they generate an optimised medication management plan for a complex multimorbid patient. Evidence of the value of collaborative decision-making between GPs to improve patient care, such as quality circles or practice-based small group learning programmes, has emerged in recent years and supports the intra-disciplinary nature of our intervention [27]. Quality circles and related groups provide an opportunity for reflective practice and discussion of troubling or challenging patient cases between GPs. Evidence shows that these groups can reduce medication costs, improve the prescribing of generic medications [27–29], and provide social support and protection against GP burnout [30]. In an exploration of cases brought by GPs to such a programme, many related to complexities in the management of patients with multimorbidity [30].

### Strengths and limitations

We developed this implementation intervention in line with the guidance issued by the MRC. Although the MRC advocates the use of the theory in the development of complex interventions [13], some researchers have questioned the usefulness of theory in this process [31, 32]. In our experience, applying theory to bottom-up data has led to an intervention which fits appropriately with existing practice and has shown real potential to change GPs’ prescribing behaviour.

To address pre-existing uncertainties associated with MY COMRADE, we used the framework method together with the implementation outcome framework [19, 20]. Framework analysis was specifically designed for data analysis in health services research, making it an appropriate choice for this work [21].

For practical reasons, we relied on self-report in the evaluation. While self-report may not be as accurate as direct observation, the interviews were conducted as soon as possible after the reviews to improve GPs’ recall and reliability [33]. Furthermore, many implementation outcomes may be best assessed using participants’ expressed attitudes and opinions, intentions or reported behaviours [19, 20].

We used our sample to purposively explore implementation in diverse practice contexts rather than to be statistically representative. The GPs who volunteered for this study are more likely to be early innovators or adopters of any improvement intervention. However, the characteristics of participating practices improve the generalisability of our results, something that implementation studies are frequently criticised for [20, 32].

While the qualitative findings from the feasibility study suggest that MY COMRADE has the potential to impact on referrals to secondary care, inappropriate prescribing and improve metrics of chronic disease care, the full scope and magnitude of effect associated with this approach has yet to be determined. Additionally, while the feasibility study addressed key uncertainties relating to the intervention’s acceptability, it did not test all uncertainties which must be answered prior to proceeding with a definitive trial of effectiveness of MY COMRADE. A pilot randomised controlled trial is now required to answer these uncertainties which include: choosing the most appropriate means for recruitment and randomisation of practices and patients; determining the time and staff requirements at the level of participating practices and the level of the research team; clarifying means of data collection from practices and patients; answering questions regarding the expected level of correlation within practices and choosing the most appropriate primary outcome for the definitive trial. Feasibility studies may be followed by pilot studies [17]; in this case, we feel such linear progression is warranted and will ultimately enhance the rigour and efficiency of the definitive trial.

## Conclusions

The MY COMRADE implementation intervention is a response to the call for interventions to support medication management in patients with multimorbidity. The intervention utilises protected time and peer support to facilitate structured medication review by GPs and generate an optimised medication management plan for a complex multimorbid patient. In this feasibility study, we found that MY COMRADE is acceptable to GPs, is adaptable to individual general practices and consistently leads to the generation of recommendations for medication optimisation. These findings suggest that MY COMRADE has the potential to make a significant contribution in improving clinical outcomes for patients with multimorbidity and justify the conduct of a larger scale trial of the intervention’s effectiveness.

## Additional files

Additional file 1: GP participant information leaflet on a feasibility study on collaborative medication review for multimorbidity in primary care. (DOCX 22 kb)
Additional file 2: Feasibility study on collaborative medication review for multimorbidity in primary care. (DOCX 19 kb)
Additional file 3: Collaborative medication review. (DOCX 25 kb)
Additional file 4: Topic guide for evaluation interviews. (DOCX 12 kb)

## Abbreviations

CPD: Continuing professional development
GP: General practitioner
MRC: Medical Research Council
MY COMRADE: MultimorbiditY COllaborative Medication Review And DEcision Making

## References

[1] Starfield B, Shi L, Macinko J (2005). Contribution of primary care to health systems and health. Milbank Q.

[2] van den Akker M, Buntinx F, Metsemakers JF, Roos S, Knottnerus JA (1998). Multimorbidity in general practice: prevalence, incidence, and determinants of co-occurring chronic and recurrent diseases. J Clin Epidemiol.

[3] Payne RA, Avery AJ, Duerden M, Saunders CL, Simpson CR, Abel GA (2014). Prevalence of polypharmacy in a Scottish primary care population. Eur J Clin Pharmacol.

[4] Galvin R, Moriarty F, Cousins G, Cahir C, Motterlini N, Bradley M, Hughes CM, Bennett K, Smith SM, Fahey T (2014). Prevalence of potentially inappropriate prescribing and prescribing omissions in older Irish adults: findings from The Irish LongituDinal Study on Ageing study (TILDA). Eur J Clin Pharmacol.

[5] Duerden M, Avery A, Payne R (2013). Polypharmacy and medicines optimisation. Making it safe and sound.

[6] Luijks HD, Loeffen MJW, Lagro-Janssen AL, van Weel C, Lucassen PL, Schermer TR (2012). GPs' considerations in multimorbidity management: a qualitative study. Br J Gen Pract.

[7] Bower P, Macdonald W, Harkness E, Gask L, Kendrick T, Valderas JM, Dickens C, Blakeman T, Sibbald B (2011). Multimorbidity, service organization and clinical decision making in primary care: a qualitative study. Fam Pract.

[8] American Geriatrics Society Expert Panel on the Care of Older Adults with M (2012). Patient-centered care for older adults with multiple chronic conditions: a stepwise approach from the American Geriatrics Society: American Geriatrics Society Expert Panel on the Care of Older Adults with Multimorbidity. J Am Geriatr Soc.

[9] Patterson SM, Cadogan CA, Kerse N, Cardwell CR, Bradley MC, Ryan C, Hughes C (2014). Interventions to improve the appropriate use of polypharmacy for older people. Cochrane Database Syst Rev.

[10] Spinewine A, Schmader KE, Barber N, Hughes C, Lapane KL, Swine C, Hanlon JT (2007). Appropriate prescribing in elderly people: how well can it be measured and optimised?. Lancet.

[11] Fraccaro P, Arguello Casteleiro M, Ainsworth J, Buchan I (2015). Adoption of clinical decision support in multimorbidity: a systematic review. JMIR Med Informatics.

[12] French SD, Green SE, O'Connor DA, McKenzie JE, Francis JJ, Michie S, Buchbinder R, Schattner P, Spike N, Grimshaw JM (2012). Developing theory-informed behaviour change interventions to implement evidence into practice: a systematic approach using the Theoretical Domains Framework. Implement Sci.

[13] Craig P, Dieppe P, Macintyre S, Michie S, Nazareth I, Petticrew M (2008). Developing and evaluating complex interventions: the new Medical Research Council guidance. BMJ.

[14] Sinnott C, McHugh S, Browne J, Bradley C (2013). GPs’ perspectives on the management of patients with multimorbidity: systematic review and synthesis of qualitative research. BMJ Open.

[15] Sinnott C, McHugh S, Boyce MB, Bradley CP (2015). What to give the patient who has everything? A qualitative study of prescribing for multimorbidity in primary care. Br J Gen Pract.

[16] Sinnott C, Mercer SW, Payne RA, Duerden M, Bradley CP, Byrne M (2015). Improving medication management in multimorbidity: development of the MultimorbiditY COllaborative Medication Review And DEcision Making (MY COMRADE) intervention using the Behaviour Change Wheel. Implement Sci.

[17] Eldridge SM, Lancaster GA, Campbell MJ, Thabane L, Hopewell S, Coleman CL, Bond CM (2016). Defining feasibility and pilot studies in preparation for randomised controlled trials: development of a conceptual framework. PLoS ONE.

[18] Lewis T (2004). Using the NO TEARS tool for medication review. BMJ.

[19] Proctor E, Silmere H, Raghavan R, Hovmand P, Aarons G, Bunger A, Griffey R, Hensley M (2011). Outcomes for implementation research: conceptual distinctions, measurement challenges, and research agenda. Admin Pol Ment Health.

[20] Peters DH, Adam T, Alonge O, Agyepong IA, Tran N. Implementation research: what it is and how to do it. BMJ. 2013;20;347:f6753. 10.1136/bmj.f6753 24259324

[21] Ritchie JL, Jane A (2003). Qualitative research practice: a guide for social science students and researchers.

[22] Hawe P, Shiell A, Riley T (2004). Complex interventions: how “out of control” can a randomised controlled trial be?. BMJ.

[23] Coiera E (2011). Why system inertia makes health reform so difficult. BMJ.

[24] Michie S, Johnston M (2012). Theories and techniques of behaviour change: developing a cumulative science of behaviour change. Health Psychol Rev.

[25] Reeve E, Shakib S, Hendrix I, Roberts MS, Wiese MD (2014). Review of deprescribing processes and development of an evidence-based, patient-centred deprescribing process. Br J Clin Pharmacol.

[26] Greenhalgh T, Howick J, Maskrey N. Evidence based medicine: a movement in crisis? BMJ. 2014;348:g3725. 10.1136/bmj.g3725 PMC4056639 24927763

[27] Wensing M, Broge B, Riens B, Kaufmann-Kolle P, Akkermans R, Grol R, Szecsenyi J (2009). Quality circles to improve prescribing of primary care physicians. Three comparative studies. Pharmacoepidemiol Drug Saf.

[28] Riou F, Piette C, Durand G, Chaperon J (2007). Results of a 12-month quality-circle prescribing improvement programme for GPs. Br J Gen Pract.

[29] Schneider A, Wensing M, Biessecker K, Quinzler R, Kaufmann-Kolle P, Szecsenyi J (2008). Impact of quality circles for improvement of asthma care: results of a randomized controlled trial. J Eval Clin Pract.

[30] Sommers LS, Morgan L, Johnson L, Yatabe K (2007). Practice inquiry: clinical uncertainty as a focus for small-group learning and practice improvement. J Gen Intern Med.

[31] Oxman AD, Fretheim A, Flottorp S (2005). The OFF theory of research utilization. J Clin Epidemiol.

[32] Bhattacharyya O, Reeves S, Garfinkel S, Zwarenstein M (2006). Designing theoretically-informed implementation interventions: fine in theory, but evidence of effectiveness in practice is needed. Implement Sci.

[33] Grol R, Wensing M, Eccles M, Davis D. Improving patient care: the implementation of change in health care. Oxford: Wiley; 2013.

