# Supplementary material for: Improving medication management for patients with multimorbidity in primary care: a qualitative feasibility study of the MY COMRADE implementation intervention
Source: Pilot Feasibility Stud. 2017 Mar 20;3:14. doi: 10.1186/s40814-017-0129-8 (PMC5357807; doi:10.1186/s40814-017-0129-8)
Supplement: Additional file 1: — GP participant information leaflet on a feasibility study on collaborative medication review for multimorbidity in primary care. (DOCX 22 kb) [file 40814_2017_129_MOESM1_ESM.docx]

**GP participant information leaflet on a feasibility study on collaborative medication review for multimorbidity in primary care.**

***Why is this study being done?***

Many patients attending GPs have multi-morbidity (multiple chronic diseases). However, clinical guidelines generally do not take multi-morbidity into account. This can lead to a situation where the guidelines for one condition suggest medications that may adversely affect a co-existing disease, or can lead to high numbers of medications or problematic polypharmacy. We have studied how GPs make decisions in these challenging multimorbid patients, and found that they often speak to their GP colleagues. In this study, we would like to formalize this interaction – that is examine what happens when two GPs review a patient’s notes together, with a view to making recommendations on the patient’s medication regimen.

**Who is organising and funding the study?**

The project is sponsored by the Health Research Board and the Health Service Executive. The research team is based in University College Cork. The principal researcher, Dr Carol Sinnott, is a trainee in General Practice. The principal investigator and supervisor is Professor Colin Bradley.

**Why am I being asked to take part?**

We are asking you to take part because in the course of your everyday work, you are likely to be faced long and complicated prescriptions for patients with multiple morbidities. We want to explore how useful a new approach to medication review would be for these cases. This new approach involves two GPs reviewing the medications together with the help of a list of prompts/checklist.

**How will the study be carried out?**

This is a feasibility study. If you agree to participate, Carol will attend your practice at a time that suits and explain how the case review should take place- this meeting will only take 15 minutes.

You will be given the checklist (includes only 7 prompting questions), which you and your GP colleague can refer to when you are systematically reviewing your patient’s medications. For the purposes of case review, we will ask you to choose 3 -5 cases from your practice, each with multiple chronic diseases that require 10+ medications or 5+ medications with another complicating factor. The case reviews, which can take place at a time that suits you and your GP colleague, will take approximately 10 minutes per case. You can make a note of any potential changes to medications on the checklist page, and scan it into the patient’s notes. This will make the next review easier and is important medico-legally. Any potential changes to medications should be discussed with the patient before making the change.

After you have completed the case reviews, Carol will re-attend your practice to explore how the process went, if any changes were made to the patient’s medications and if you have any recommendations on how it could be improved. We will not ask for any patient identifying information. However we will take details of the patient’s age, gender, diagnoses and list of medications. With your permission, we will record this second meeting, and the recorded data will be analysed for recurrent issues that arise for GPs in this area.

***What about confidentiality?***

All information obtained during the study will be strictly confidential. All identifiable information will be removed from recorded data. A study ID number will be assigned to any data relating to your practice, to maintain anonymity. Only investigators named on this information sheet will have access to the data, which will be stored securely in UCC.

***What will happen with the results of this study?***

The findings of this study will be written up for the HRB report and subsequent publications. The results will also be compiled and submitted as part of a PhD thesis. In all cases only anonymous extracts or quotes will be reported. Copies of the findings will be made available to participants.

***Who has reviewed this study?***

This research gained approval from the ethics committee of the CREC, UCC. If you decide to take part you will be asked to sign a consent form. You are free to withdraw at any time. If you have any questions or concerns, please do not hesitate to contact the research team detailed below.

| Dr Carol Sinnott | Professor Colin Bradley |
| --- | --- |
| GP Trainee, South East Training Scheme | Professor and Head of Department |
| Research Fellow, University College Cork | Department of General Practice, University College Cork. |
| [csinnott@ucc.ie](mailto:csinnott@ucc.ie) | [gp@ucc.ie](mailto:gp@ucc.ie) |
| 021 4205527 | 021 4901572 |

***Thank you for taking the time to read this information.***
