# Supplementary material for: Improving medication management for patients with multimorbidity in primary care: a qualitative feasibility study of the MY COMRADE implementation intervention
Source: Pilot Feasibility Stud. 2017 Mar 20;3:14. doi: 10.1186/s40814-017-0129-8 (PMC5357807; doi:10.1186/s40814-017-0129-8)
Supplement: Additional file 2: — Feasibility study on collaborative medication review for multimorbidity in primary care. (DOCX 19 kb) [file 40814_2017_129_MOESM2_ESM.docx]

**Feasibility study on collaborative medication review for multimorbidity in primary care**

What to do

1. Choose 3 patients each on which to do the medication review. Try to choose patients prescribed 10+ medications or 5+ medications with another complicating factor.
2. Schedule a time to discuss these patients with another GP in your practice
3. Use the attached checklist as a guide for the discussion. Make a note of any potential changes to medications on the page, and scan into the patient’s notes.
4. Please try to complete the cases reviews within the next month – they take approximately 10mins each, but may take longer initially.

Before starting, Consider

1. What benefits would you see in this format for medication review? What might make it difficult?
2. What plan would suit *your practice*, for trying this out?

- What day of the week?
- What time of day?
- Which office?
- How many case reviews will you do at one sitting?
- Which GP will you involve?
- Anything else, specific to how your practice runs?

Additional points

- Document the medication review in the patient’s notes - it will make the next review easier and is important medico-legally.
- Highlight any potential options for medication changes -these options should be discussed with the patient at their next consultation, prior to making any changes.
- Internal CME points apply.
- For further information, please contact: Dr Carol Sinnott, Research fellow in General Practice, UCC. csinnott@ucc.ie
