# Supplementary material for: Improving medication management for patients with multimorbidity in primary care: a qualitative feasibility study of the MY COMRADE implementation intervention
Source: Pilot Feasibility Stud. 2017 Mar 20;3:14. doi: 10.1186/s40814-017-0129-8 (PMC5357807; doi:10.1186/s40814-017-0129-8)
Supplement: Additional file 3: — Collaborative medication review. (DOCX 25 kb) [file 40814_2017_129_MOESM3_ESM.docx]

**Collaborative Medication Review**

Review by: Dr. _________________ & Dr. ___________________

Date of review:

Patient name & DOB:

- Give your GP colleague a brief description of the case (e.g. 75yearold lady, lives alone, history of diabetes and arthritis).
- Discuss each medication (or groups of medications, such as anti-hypertensives) using the points below.
- Not all points will be relevant.

| **N** | What is the **need** or indication for this medication? |
| --- | --- |
| **O** | Is this need **on-going**? Has the patient’s condition or life expectancy changed since this medication was started? Was long term treatment intended? |
| **T** | Is the patient getting appropriate **tests** and monitoring associated with this medication? |
| **E** | Has the **evidence** or guidelines changed in relation to this medication/ condition since it was commenced? (*think of big messages*) |
| **A** | Are there any **adverse effects** with this medication? Check for interactions, duplications, contraindications. |
| **R** | **Risk reduction** and prevention: Are doses/ medications optimised to lower the patient’s risk? |
| **S** | Can treatment be **simplified** to a safer /easier to use alternative? |

List the medications where there is potential to change / further action required:

Additional points

- The medication review should be documented in the patient’s notes (e.g. scan in this page). It will make the next review easier.
- Any options for medication changes should be discussed with the patient at their next consultation, prior to making any changes.
- The ‘NO TEARS’ checklist is adapted from the BMJ 2004;329:434
