# Supplementary material for: Improving medication management for patients with multimorbidity in primary care: a qualitative feasibility study of the MY COMRADE implementation intervention
Source: Pilot Feasibility Stud. 2017 Mar 20;3:14. doi: 10.1186/s40814-017-0129-8 (PMC5357807; doi:10.1186/s40814-017-0129-8)
Supplement: Additional file 4: — Topic guide for evaluation interviews. (DOCX 12 kb) [file 40814_2017_129_MOESM4_ESM.docx]

**Topic guide for evaluation interviews**

1. Acceptability: what was your initial impression of this approach to MR? Were you optimistic that it would work?* Is it credible, does it have any advantages to existing approaches.

2. Adoption: what was your initial plan on how to bring this into your practice?* What were your goals and intentions with relation to it?*

3. Appropriateness: how fitting is this intervention to the setting of multimorbidity? How fitting is it to the setting of GP? Did you think it would be useful? For what?* How was making decisions in this format (easier or more complicated)?* What about discussing your practice with another GP – comfortable / uncomfortable?

4. Feasibility: how feasible is it for you right now, to continue doing this? Is it practical / trialable? Were you confident that you could conduct it? Any concerns about being able to do it? * what are the main barriers ( need link to meds info for example?)

5. Fidelity: how did you do it? What happened in the review?

Especially specific BCTs: social support, checklist – addition of it to review/ content of, action planning, changing social environment, awarded CME points?

Features most related to success/failure of intervention?

6. Implementation cost: were there opportunity costs? Were there other things that you could not do as a result of this intervention? (ie house visits / seeing patients / going home)

7. Coverage: how widely applicable is this to your patients on multiple meds? Are there many that you find this not appropriate in?

8. Sustainability: do you think it could become routine. Incorporated in to regular practice? What are the incentives for you to continue (CME/safety/ time saver)?*
